# European guidelines for personality disorders: past, present and future

**DOI:** 10.1186/s40479-019-0106-3

**Published:** 2019-05-21

**Authors:** Sebastian Simonsen, Anthony Bateman, Martin Bohus, Henk Jan Dalewijk, Stephan Doering, Andres Kaera, Paul Moran, Babette Renneberg, Joaquim Soler Ribaudi, Svenja Taubner, Theresa Wilberg, Lars Mehlum

**Affiliations:** 1 Stolpegaard Psychotherapy Centre, Copenhagen, Denmark; 2 University College, London and Anna Freud National Centre for Children and Families, University of Copenhagen, London, UK; 3 Institute of Psychiatric and Psychosomatic Psychotherapy; Central Institute of Mental Health, Heidelberg University, Mannheim, Germany; 4 Private practice, Weesp, The Netherlands; 5 Department of Psychoanalysis and Psychotherapy, Medical University of Vienna, Vienna, Austria; 6 Kanta-Häme Central Hospital, Hämeenlinna, Finland; 7 Centre for Academic Mental Health, Department of Population Health Sciences, Bristol Medical School, University of Bristol, Bristol, UK; 8 Freie Universität, Berlin, Germany; 9 Department of Psychiatry, Hospital de la Santa Creu i Sant Pau, Barcelona, Spain; 10 Centro de Investigación Biomédica en Red de Salud Mental, CIBERSAM, Madrid, Spain; 11 Universitätsklinikum Heidelberg | Universität Heidelberg, Heidelberg, Germany; 12 Department of Research and Development, Division of Mental Health and Addiction, Oslo University Hospital and Institute of Clinical Medicine, University of Oslo, Oslo, Norway; 13 National Centre for Suicide Research and Prevention, Institute of Clinical Medicine, University of Oslo, Oslo, Norway

**Keywords:** Guidelines, Personality disorders, Recommendations

## Abstract

Personality disorders (PD) are common and burdensome mental disorders. The treatment of individuals with PD represents one of the more challenging areas in the field of mental health and health care providers need evidence-based recommendations to best support patients with PDs. Clinical guidelines serve this purpose and are formulated by expert consensus and/or systematic reviews of the current evidence. In this review, European guidelines for the treatment of PDs are summarized and evaluated. To date, eight countries in Europe have developed and published guidelines that differ in quality with regard to recency and completeness, transparency of methods, combination of expert knowledge with empirical data, and patient/service user involvement. Five of the guidelines are about Borderline personality disorder (BPD), one is about antisocial personality disorder and three concern PD in general. After evaluating the methodological quality of the nine European guidelines from eight countries, results in the domains of diagnosis, psychotherapy and pharmacological treatment of PD are discussed. Our comparison of guidelines reveals important contradictions between recommendations in relation to diagnosis, length and setting of treatment, as well as the use of pharmacological treatment. All the guidelines recommend psychotherapy as the treatment of first choice. Future guidelines should rigorously follow internationally accepted methodology and should more systematically include the views of patients and users.

## Introduction

Individuals with PD often suffer extreme distress and social impairment. Compared to those without a PD, they lead shorter lives, and their quality of life is often significantly reduced [1, 2]. Treatment providers for patients with PDs face a difficult task and need guidance from robust evidence in order to meet these challenges. Thus, across the world, health authorities have independently developed clinical guidelines for management of people with PDs. Clinical guidelines are systematically developed expert statements to assist practitioner and patient decisions regarding appropriate healthcare for specific clinical circumstances [3]. The first systematically developed European guidelines on the management of PDs were published in 2008 and 2009 and came from Finland, Germany, the Netherlands and the UK. Furthermore, a widening gap between healthcare costs and increasing public demand for high-quality services has stimulated interest on the need for robust guidelines in order to guide more efficient targeting of resources [4, 5]. Although costs are contextually determined to some extent, it does seem that there are some similarities in health systems and costs across European countries, and one can therefore argue that a European perspective on the treatment of PDs might exist and that this should be reflected in the guidelines as well.

Guidelines vary in methodological approach and quality, and most guidelines differentiate between evidence and practice-based recommendations. Guideline quality can be assessed and quantified using the AGREE system (Appraisal of Guidelines, Research and Evaluation), where a guideline is assessed across six domains [6]. See Table 1 for domains. An important issue when developing guidelines is how to arrive at recommendations based on a systematic method. The Grade working group (www.gradeworkinggroup.org) provides guidance on how to link the body of evidence with the degree of strength of the recommendation (strong vs. weak/conditional). GRADE emphasizes the importance of phrasing recommendations using active language and of avoiding ambiguous or unclear wording, such as ‘if clinically appropriate’ or ‘if necessary’ [7]. Guidelines using specific and active language have been shown to lead to greater adherence than guidelines using vague or nonspecific phrasing [8]. Thus, it can be a fine balance for guideline developers not to go beyond the evidence and yet still provide guidance that is sufficiently specific, practical and clinically useful.

**Table 1:** Domains in AGREE II

Domain	Main content
Scope and purpose	Objectives, population and clinical questions have been clearly described.
Stakeholder involvement	The guideline development group includes all relevant professional groups, and patients’ views and preferences have been included in the process.
Rigour of development	Systematic search and use of evidence and link between evidence and recommendations. Guideline has undergone external review prior to publication
Clarity of presentation	Recommendations are specific and unambiguous and easily identified.
Applicability	Potential organizational barriers (including costs) are discussed, and key review criteria for monitoring and audit are provided.
Editorial independence	The editorial process is independent from the funding body, and any conflicts of interests are disclosed.

In this paper we provide an overview of the European guidelines that we are aware of and highlight key recommendations for improvement. For each guideline we considered the AGREE domains and especially the rigour in the development and applicability of the guidelines. In reviewing the guidelines, ultimately our aim was to identify areas of convergence as well as divergence with a view to assisting the refinement of future iterations of guidelines.

### Organizational background of European guidelines

We first provide a general overview of identified guidelines based on geographical location and with emphasis on organizational background, rigour of development and perceived applicability.

### Northern Europe

The earliest European guidelines that we were able to identify derived from The Swedish Psychiatric Society. The Society developed *Clinical guidelines for personality disorders* in 2006, which were recently updated (2017) [9].

#### Rigour of development

The current, revised clinical guidelines were developed based on existing research evidence. They do not include a method section outlining a systematic evaluation of the empirical support underlying different recommendations, but rather rely heavily on guidelines from other countries, e.g., The National Institute for Health and Care Excellence [10] and the Australian Clinical Practice Guidelines [11]. On the other hand, strengths and shortcomings in current evidence, as well as topics for further research, are integrated aspects of the clinically-oriented discussion of care. From an evidence-based guidelines perspective, the main limitation concerns the rigour of development, more specifically that the path from evidence to recommendation is not always transparent and reproducible.

#### Applicability

the goal of the Swedish guidelines appears to have been to develop guidelines with a ‘clinical foundation’, focusing on the daily work with patients and their families. The guideline takes into account a range of clinical issues regarding the evaluation and treatment of patients with PDs at different levels of healthcare. It discusses dynamics and requirements at an organizational level as well as the need for interventions and care at the community level, including work support.

The Finnish Current Care Guidelines for BPD were developed by the Finnish Medical Society Duodecim in cooperation with the Finnish Psychiatric Association in 2015 [12]. This is the second version of the guidelines.

#### Rigour of development

The Finnish Medical Society Duodecim is one of the founding members of the Guidelines International Network (G-I-N), so the BPD guideline has been assessed according to the G-I-N guideline standards [13]. Working groups consisted of leading volunteer healthcare professionals as ‘content experts’ and Current Care editors as ‘method experts’. Before final edits and publishing, the guidelines were sent to relevant interest groups, including patient representatives, for comments. Systematic method and evidence reviews were applied in the development process, but the guideline recommendations can only partly be traced back to the supporting evidence.

#### Applicability

The guidelines are intended to be used by physicians and healthcare professionals, and therefore focus on questions relating to diagnosis, psychotherapy in general and are somewhat more specific with regard to medication. They are made publicly available (http://www.kaypahoito.fi/web/kh/suositukset/suositus?id=hoi50064), and patient versions are available for use by the public.

In Denmark, the National Health Authority published guidelines for BPD in 2016 [14].

#### Rigour of development

The working group consisted of members from several professional associations and of method consultants from the National Health Authority. In addition, the working group was overseen by a reference group consisting of experts, consumers and individuals in senior management positions. The GRADE system was used in developing the recommendations. The working process, methods and analyses behind the recommendations are publicly available on the National Health Authority website (https://www.sst.dk/da/udgivelser/2015/nkr-borderline), and include an English quick-guide translation.

#### Applicability

Ten specific questions with regards to screening in primary care, diagnosis, length of treatment, uni- or multimodality treatment, monitoring outcomes and pharmacological treatment formed the starting point for the development of the guideline. The guidelines have been criticised for lack of applicability by clinicians and administrators e.g. how is it helpful or practical to know that there is not robust evidence for differences in outcomes between short vs. long treatments? The Danish guidelines are currently in the process of being updated.

Currently, there are no Norwegian national clinical guidelines for PDs. The Norwegian National Advisory Unit on Personality Psychiatry (NAPP) has recently sent an inquiry to the Norwegian health authorities recommending the development of national clinical guidelines to stimulate the establishment of treatment programmes, sound evaluation practices and adequate referral criteria to various levels of the healthcare system.

### Western Europe

In Germany, the AWMF (Arbeitsgemeinschaft der Wissenschaftlichen Medizinischen Fachgesellschaften, in English: The Association of the Scientific Medical Societies in Germany) is responsible for the development of guidelines by the scientific medical societies.

#### Rigour of development

Treatment guidelines for PD were first developed in 2009 by a committee of experts as delegated by a variety of professional societies and associations [15]. A systematic search for evidence was conducted, and the body of evidence is well described. The AWMF distinguishes between different levels of guidelines based on their level of evidence and quality, from S1 (experts’ recommendations) to S3 (systematic and evidence-based) [16]. The first guideline was at level S2. Currently, an S3 guideline on BPD is in progress, and strong emphasis has been placed on maximum transparency and applicability in addition to systematic integration of both evidence and consensus.

#### Applicability

Due to the lack of efficacy studies, with the exception of BPD, recommendations in the 2009 guideline for the treatment of PD subtypes are based primarily on clinical expertise or published expert opinions. This is a major limitation as guidelines based on consensus are likely to have limited impact.

In the Netherlands, the first Multidisciplinary Guideline for PD was published in 2008 [17].

#### Rigour of development

The guideline was developed by an expert workgroup and is based on the results of scientific research critically and systematically appraised according to the level of evidence and completed with professional expertise. However, the connection between evidence and strength of recommendations is not fully transparent and many recommendations were based on very low levels of evidence.

#### Applicability

The guideline was developed to improve the treatment of individuals with PDs. Questions concerning the role of the patient and the family, diagnosis, therapeutic interventions, nursing care, vocational therapies, pharmacological interventions, co-morbidity, cost effectiveness and organization of care, guided the discussions of a Working Group that was responsible for the result. The major limitation of the guideline concerned implementation, especially in regard to systematically considering barriers and monitoring progress. Furthermore, the guideline has been criticized for being too focused on patients receiving specialist psychotherapy and neglectful of other types of care e.g. psychiatric management and vocational therapy. This perspective has now been covered in a standardized procedure of care for patients with BPD published in 2017 [18]. This Standard of Care was developed and formulated from the patient’s perspective and the main goal of care was psychosocial recovery.

In the United Kingdom, National Institute of Health Care and Excellence (NICE) is an independent public body that provides national guidance and advice to improve health and social care in the United Kingdom (https://www.nice.org.uk/). NICE commissions the National Collaborating Centre for Mental Health to set up working groups to develop guidelines. In 2009, the Guidelines on BPD [10] and Antisocial Personality disorder [19] were published.

#### Rigour of development

The guidelines cover many clinically important questions, but recommendations are often based on clinical/expert consensus rather than evidence. The emergence of new evidence has been monitored since 2009, but none of the emergent evidence has been deemed sufficiently strong to warrant changing the guideline recommendations.

#### Applicability

Despite criticism of some of the recommendations [20], the guidelines have been highly influential. Because of the proficiency in English by most academics across Europe, these guidelines have been used and referenced in all later European guidelines, and according to Google Scholar, the two guidelines have thus far been cited more than 250 times in the scientific literature.

In Switzerland, the Swiss Association for Psychiatry and Psychotherapy set up a task force consisting of personality experts with the aim of coming up with practical and relevant clinical treatment recommendations for BPD rather than a new guideline. The task force published recommendations in 2018 (https://www.psychiatrie.ch/sspp/specialistes-et-commissions/recommandations-therapeutiques) [21]. The recommendations are currently available in French and German.

#### Rigour of development

The guideline has several strengths in addressing important clinical questions and in considering national particularities but may be criticised in terms of a lack of transparency in using multiple levels of evidence without defining how this is reflected in the strength of recommendations.

#### Applicability

Recommendations were based on a consensus view giving weight to scientific evidence, good clinical practice and national applicability. However, a lack of diverse representation in the guideline development group may have limited the utility of these recommendations.

### Southern Europe

In Spain, a clinical practice guideline for BPD was developed in 2011 by the Catalan Agency for Health Information, Assessment and Quality.

#### Rigour of development

Methodologically, the guideline was developed using the system SIGN (Scottish Intercollegiate Guidelines Network) [22]. The guideline was developed by a team including a methodology consultant, psychiatrists, psychologists, a nurse and social workers, and was based on a systematic search and rating (AGREE and OSTEBA) of evidence.

#### Applicability

The guideline is oriented towards specialists in mental health who are responsible for the treatment and care of individuals with BPD, including: psychiatrists, clinical psychologists, nurses, social workers, educators, occupational therapists, and other professionals of the NHS. Clinical areas included in the guideline are: prevention, diagnosis and interventions in both psychological and psycho-social domains as well as in pharmacological treatment, organization of services and programs for primary health care services, community care services, hospitalization services, partial hospitalization services / day hospital, community rehabilitation services, continuation of care program, and care itinerary. Issues pertaining to applicability and implementation were, similarly to other guidelines, only addressed sparsely.

### Main recommendations across guidelines

In evaluating the different guidelines, we focused on three main areas: diagnosis, psychological treatment and pharmacological treatment. These areas were chosen because recommendations in diagnosis and general treatment should be independent of national health systems and thus be generalizable to the whole of Europe. It is clear that there are both similarities and discrepancies across guidelines.

Regarding diagnoses (see Table 2), there is some consensus on the use of semi-structured interviews, although the Swedish guidelines specifically state that such interviews alone are not sufficient. Instead, they suggest adopting the LEAD principle (Longitudinal Expert All Data) as the gold standard. Screening tools are addressed in three guidelines. The Swedish guidelines warn against using screening for diagnostic purposes, while the Danish guidelines go even further in not recommending the use of screening in primary care due to the high rate of false positives and negatives. Finally, the Swiss guidelines recommend the use of screening instruments to ascertain specific symptoms and differential diagnoses. The question of severity of the disorder is only directly addressed in the new Swiss guidelines, which state that it should be taken into consideration. On a general level, the consensus regarding the diagnosis of PD is focused on which instruments to use and with what purpose and caveats. Only the Swedish guideline recommends that while an evaluation of general criteria can be conducted in any part of the healthcare system, the diagnosis of a specific personality syndrome should only be made by specialist psychiatric services. It is not entirely clear whether this runs counter to the British guideline, which specifies that community mental health services should be responsible for routine assessment.

**Table 2:** European recommendations on PD diagnoses

Guideline	population	Recommendations
Swiss (2018)	BPD	BPD is diagnosed according to the ICD-10 (11) or DSM-5 criteria and a structured interview (e.g., SCID-II, IPDE) is recommended for the final diagnosis. The dimensional depiction of the psychosocial severity is gaining importance for the treatment plan and should be taken into consideration, e.g., according to criterion A in DSM-5. Differential diagnoses of BPD should be carefully distinguished. Specific symptoms and differential diagnoses can be additionally ascertained with screening instruments (e.g., questionnaires)
Swedish (2017)	PD	Screening tools, self-report or semi-structured diagnostic interviews are not sufficient for diagnosis. Diagnostic evaluation should be based on the LEAD principles (Longitudinal Expert All Data). Evaluation of general criteria for personality syndrome can be made in all parts of the health care system, while diagnosing specific personality syndromes is a task for the psychiatric specialist services.
Danish (2016)	BPD	Screening tools should not be used for the identification of potential borderline personality disorder in the primary sector on a routine basis. It is good practice to diagnose patients with borderline personality disorder using a semi-structured clinical personality interview.
Finnish (2015)	BPD	SCID-II-interview may increase the accurateness of PD diagnosis.
Catalonia (2011)	BPD	It is recommended as good practice to use a semi-structured clinical personality interview for the diagnosis. Diagnosis preferably from the age of 16 to be restrictive in the diagnosis of the youngest. Make appropriate differential diagnosis to distinguish from other disorders
German (2009)	PD	Patients with PD should be diagnosed using a (semi)-structured clinical interview. For dimensional rating, disorder-specific self-assessment questionnaires are recommended. Open communication of diagnosis is recommended
British (BPD)	BPD	Community mental health services should be responsible for routine assessment.
British (2009)	ASPD	When assessing a person with possible antisocial personality disorder, fully assess: antisocial behaviours, personality functioning, coping strategies, strengths and vulnerabilities, comorbid mental disorders (including depression and anxiety, drug or alcohol misuse, post-traumatic stress disorder and other personality disorders), the need for psychological treatment, social care and support, and occupational rehabilitation or development and domestic violence and abuse. Use structured assessment methods whenever possible to increase the validity of the assessment. In forensic services, use measures such as PCL-R or PCL-SV to assess the severity of antisocial personality disorder as part of the routine assessment process.
Dutch (2008)	PD	The diagnosis of a personality disorder is preferably based on a combination of a clinical interview and structured interviews.

With regard to recommendations for psychological treatment, it is noteworthy that the vast majority of evidence and recommendations pertains to BPD (See Table 3). The only recommendations we found for Antisocial Personality Disorder were cognitive-behavioural therapy (CBT), which was recommended by both the British and the German guidelines. In addition, the German guidelines state that CBT has the strongest empirical support in the treatment of Avoidant PD (AvPD). However, both guidelines date back to 2009, with new relevant evidence having emerged since then [23, 24].

**Table 3:** European recommendations on psychotherapy for Personality disorders

Guideline	Pop.	Recommendations
Swiss (2018)	BPD	The primary form of treatment is outpatient psychotherapy 1–2 sessions a week over a time span of 1–3 years. Disorder-specific inpatient psychotherapy (In a psychotherapeutic ward with a treatment concept adapted *specifically* for BPD) (Elective treatment according to individual indication).
Swedish (2017)	PD	Treatment of personality syndromes may often involve multidisciplinary teams and multimodal programs. Specialist services should be able to offer one or more of the evidence-based psychotherapies for borderline personality disorder. There is insufficient empirical support for choosing between short-term or long-term psychotherapies.
Danish (2016)	BPD	It is good practice to offer either multimodal treatment programs including psychotherapy or unimodal psychotherapy to patients with borderline personality disorder. It is good practice to offer either short-term psychotherapy (< 12 months) or long-term psychotherapy (≥ 12 months). It is good practice to consider monitoring psychotherapy offered to patients with borderline personality disorder.
Finnish (2015)	BPD	Some psychotherapeutic approaches can effectively relieve the symptoms and distress of patients as well as promote adaptation and enhance functioning. Treatment should be delivered as outpatient treatment as much as possible, and inpatient treatment should be mostly day hospital treatment.
Catalonia (2011)	BPD	Recommend the use of DBT for treatment and (with less evidence) the use of MBT and Schema Focused Therapy
German (2009)	PD	Four treatments are recommended as good practice: dialectic-behavioral therapy (DBT), mentalisation-based therapy (MBT), schema therapy/ schema-focused and transference-focused therapy (TFP). DBT treatment shows better empirical evidence than MBT, schema-focused therapy and TFP for BPD.
British (BPD) (2009)	BPD	When providing psychological treatment for people with borderline personality disorder, especially those with multiple comorbidities and/or severe impairment, the following service characteristics should be in place: - An explicit and integrated theoretical approach used by both the treatment team and the therapist, which is shared with the service user - Structured care in accordance with this guideline - Provision for therapist supervision. - Although the frequency of psychotherapy should be adapted to the person’s needs and context of living, twice-weekly sessions may be considered. Do not use brief psychotherapeutic interventions (of less than 3 month’s duration) specifically for borderline personality disorder or for the individual symptoms of the disorder.
British (2009)	ASPD	For people with antisocial personality disorder, including those with substance misuse problems, in community and mental health services, consider offering group-based cognitive and behavioural interventions, in order to address problems such as impulsivity, interpersonal difficulties and antisocial behaviour.
Dutch (2008)	PD	Several individual ambulatory psychotherapies are effective in treating people with a personality disorder. There is evidence that therapies that have been shown effective in treating Axis I disorders without a personality disorder are also effective in treating people who also have a personality disorder. There is evidence that treating people with a personality disorder with psychotherapy is cost effective compared to treatment as usual and no therapy

For BPD, there is broad consensus that outpatient psychotherapy should be the primary treatment. However, inpatient treatment specifically adapted to BPD and day hospital treatment are also recommended. The recommendations in terms of both length of treatment and use of multiple modalities are not clear and are sometimes contradictory even within a guideline as well as between guidelines. For instance, the Danish guidelines include a practice-based recommendation of both short (< 12 months) and long-term treatments, while the British guidelines specifically warn against the use of psychotherapy treatments lasting less than 3 months. Also, few guidelines mention specific theoretical approaches, such as Mentalization-Based Therapy (MBT), Dialectical Behaviour Therapy (DBT), Transference-focused Psychotherapy (TFP), or Schema Therapy. One noteworthy exception, however, is the German guideline, which specifically state that DBT has better empirical evidence than other types of specialized psychotherapy for BPD. However, in a recent meta-analysis it was concluded that both DBT and psychodynamic approaches (the last category defined very broadly and including both MBT and brief therapy based on psychoanalytic principles) are effective for BPD symptoms [25].

Finally, recommendations on pharmacological treatment are in overall agreement that, based on sparse trial evidence, medication should not be considered the primary intervention for PD, but should be used mainly for treating comorbid disorders and in some cases used briefly during times of crisis (see Table 4). The Swiss, Finnish and Dutch guidelines suggest that medication may be used to reduce specific dimensions of BPD such as anger, impulsivity or negative mood. However, these specific recommendations are not consistent and are somewhat at odds with the more general recommendation of being cautious with the use of medications.

**Table 4:** European recommendations on medication for Personality disorders

Guideline	Pop.	Recommendations
Swiss (2018)	BPD	Medication should be restricted to critical situations and administered for a short timespan In case of need, symptom-focused hierarchical organization - Lamotrigin and Topiramat is administered for anger, aggression and impulsivity - Quetiapin and Aripiprazol is administered for irritability and cognitive-perceptive symptoms Generally, dosage is kept in the lower range. Benzodiazepines should be completely avoided Treatment of comorbidities should be evaluated systematically and thoroughly No Polypharmacy.
Swedish (2017)	PD	Medication should not be offered as a primary treatment for personality syndromes but may be applied treating co-occurring symptom disorders.
Danish (2016)	BPD	Antidepressants should only be used for the treatment of patients with borderline personality disorder upon due consideration. Mood stabilizers should only be used for the treatment of patients with borderline personality disorder upon due consideration. Antipsychotics should only be used for the treatment of patients with borderline personality disorder upon due consideration
Finnish (2015)	BPD	Antipsychotic medication might relieve symptoms in multiple dimensions. Mood stabilizers may be useful in reducing impulsivity and aggression. Serotonin reuptake inhibitors may be useful especially in treatment of comorbidity. There is a risk of polypharmacy in pharmacological treatment. Mood stabilizers and 2nd generation antipsychotics are preferred in pharmacotherapy.
Catalonia (2011)	BPD	There is no evidence for any pharmacological treatment. It is recommended to avoid the use of benzodiazepines due to the risk of abuse and dependence. The pharmacological treatment should be considered as a coadjuvant of the psychotherapeutic or the psychosocial intervention to globally improve or to improve one of its characteristic symptoms. The pharmacological treatment in patients with BPD must be periodically reviewed, with the aim of eliminating unnecessary or ineffective medications as well as avoiding polypharmacy.
German (2009)	PD	Pharmacological treatment can be considered for crisis-like aggravation and comorbid disorders. There is no evidence for pharmacological treatment of PD only, it should always be combined with psychotherapy.
British (BPD) (2009)	BPD	Do not use: Drug treatment specifically for borderline personality disorder or for the individual symptoms or behaviour associated with the disorder. Antipsychotic drugs for the medium- and long-term treatment of borderline personality disorder. Consider drug treatment in the overall treatment of comorbid conditions. Consider cautiously short-term use of sedative medication as part of the overall treatment plan for people with borderline personality disorder in a crisis. Agree the duration of treatment with them, but it should be no longer than 1 week. Review the treatment of those who do not have a diagnosed comorbid mental or physical illness and who are currently being prescribed drugs. Aim to reduce and stop unnecessary drug treatment.
British (2009)	ASPD	Pharmacological interventions should not be routinely used for the treatment of antisocial personality disorder or associated behaviours of aggression, anger and impulsivity.
Dutch (2008)	PD	There is evidence that antipsychotics, SSRI’s and mood stabilizers may improve targeted symptoms of a personality disorder and the global functioning.

## Discussion

Over the past decade, throughout Europe, a range of clinical guidelines for the management of PDs have emerged. The development of more rigorous guidelines has only been possible through the exponential growth of research data showing that PDs, particularly BPD are treatable conditions. The publication of dismantling studies, and the wider availability of treatment manuals and adherence rating scales have further assisted the process of scrutinising the process of treatment and its efficacy. These activities should instil optimism for the future development of high-quality mental health services for people in need of specific PD treatments across Europe. However, as we have shown in this brief overview, existing guidelines still have many limitations that need to be effectively tackled in future iterations.

Although national variations in the context of mental health service delivery and varying needs of the populations could justify some variation between national guidelines, the differences we have identified are probably due to, to a large extent, the lack of methodological rigour with which guidelines have been developed along with the paucity of evidence for many of the clinical questions addressed by the guidelines. Since guidelines should base their recommendations on an evidence-based paradigm, and since their strongest recommendations stem from randomized controlled trials, it would be reasonable to expect guideline authors to adopt the same strict practices required when reporting such trials (Consolidated Standards of Reporting Trials – CONSORT) (http://www.consort-statement.org). This would mean that guideline authors should adhere to harmonised standards for reporting their recommendations in a complete and transparent way. Unfortunately, currently, we are far from such practices. Although the number of RCTs has considerably increased since the development of the first BPD Guideline [26], the need for further research should be stressed, as many of the current evidence-based treatments for BPD do not yet reach the condition of “sufficient evidence”. The particular case of DBT compared to TAU, may be the only exception [27]. A second limitation seen in the majority of the guidelines reviewed, is a lack of a systematic process for capturing the views and values of patients and carers. In order to be relevant and useful, clinical guidelines need to provide clinicians with advice that will help them make decisions about patient care based, on the weighting and negotiation of medical knowledge arising from more sources than experimental research. Patients’ needs, and values are important sources of such knowledge and should be solicited in a systematic way in the process of establishing future clinical guidelines.

As our review clearly shows, clinical guidelines for PD treatment focus almost exclusively on research on BPD, although this has not always been made explicit by the authors. There is, however, a need to distinguish general PD guidance from BPD guidance. Naturally, there are similarities in the broader field of recommended PD treatment, but also important differences, for example with respect to treatment of suicidal and self-harming behaviours, where arguably distinctions in the guidance for BPD and other PDs should be made [28]. A challenge to making such clear distinctions is, of course, the widespread comorbidity often seen in people with PDs, not only in terms of several comorbid PDs, but also in terms of comorbidity with other clinical syndromes, such as anxiety, depression, post-traumatic stress disorder and substance use disorders. People with multiple disorders often receive too many or conflicting therapies and solving this problem and creating a basis for personalized treatment is a huge challenge to future development of clinical guidelines for PD treatment.

Little is gained if clinical guidelines are not made known among clinicians who need them and if no measures are taken to implement them in the field of practice. This crucial aspect of promoting evidence-based practices regarding PD treatments is seemingly neglected in existing guidelines, as we have shown. Guidelines could include steps to be taken to ensure that clinicians receive proper training in empirically supported treatments including how systems of supervision and adherence rating could be put in place to ensure that standards are upheld over time. Guideline documents could also provide guidance on how to conduct clinical audits in mental health service centres at regular intervals as a measure to assess the need for improvements on a larger scale. These are just some suggestions on how implementation aspects could be addressed in future guideline documents. Clearly, there is a need for more research to study the real effectiveness of such implementation measures. The implementation of empirically-based treatments and its empirical documentation is lagging at least two decades behind the development and empirical validation of the treatments themselves [29].

The recently published DSM-5, with its Alternative Model for PDs as well as the upcoming ICD-11, focuses a great deal on severity in the diagnosis of PDs. With the notable exception of the recent Swiss guideline, none of the guidelines address the issue of severity. There are empirical data showing that more severely disturbed patients might need longer treatments to improve [30], as well as population-based data showing that severity of PD is robustly associated with future risk of poor health and relationship difficulties [31] . Furthermore, a clinically derived stepwise model on how to apply different and more intense treatments for BPD patients with increasing severity has recently been published by [32]. In light of prominence in both ICD-11 and DSM-5, we think the issue of severity will have to be considered in future iterations of guidelines.

Although we found a considerable overlap between the different national guidelines, there were also significant differences. These may in part reflect the different ages of the guidelines. In addition, different domains of outcome are now looked at more thoroughly today than they were in the past and it is acknowledged that a treatment that is effective in one domain does not necessarily confer effective treatment in another domain [26]. Another discrepancy in the guidelines´ recommendations can be found regarding medication. While for example, some guidelines recommend the prescription of antipsychotic or antidepressant medication to target specific symptoms, other guidelines insist on a general caution when it comes to psychopharmacological treatment. Whilst there is no current evidence that any form of medication can modify the enduring features of personality, there is some evidence for positive effects of medication in the domains of mood stabilization and impulsivity [33]. As a consequence, future guidelines should – as mentioned above – clarify the rationale of their recommendations in terms of the target domains of different treatments.

Finally, the expiry date of guidelines warrants consideration. We think in view of the rapidly growing number of empirical studies in the field, the relevance and completeness of BPD guidelines should be reviewed at least every 5 years. Following outdated guideline recommendations might not reflect empirically-based treatment, but rather the opposite. Thus, we strongly support the practice of the German Association of the Scientific Medical Societies, that remove a guideline from its website five years after publication unless it is updated according to a defined procedure. For PDs in general a five years criterion might be too strict due to the slower accumulation of evidence.

As a final methodological consideration of this overview of European guidelines we acknowledge that use of the AGREE system would have been a more systematic approach for guideline quality appraisal. However, the AGREE manual [6] recommends at least 2 appraisers and preferably 4 in order to achieve a reliable rating and due to language barriers and the unfunded nature of the project this was not considered feasible.

## Conclusions

We conclude that a) a more systematic approach of capturing the views and values of patients and carers is needed in the process of developing new guidelines; this will help make the guidelines more clinically relevant and have greater utility of clinicians and patients alike, b) since guidelines have so far focused almost exclusively on BPD and guidelines for other PDs are either outdated or missing completely, there is a strong need for future guidelines to include these other PDs, c) future guidelines need to have a stronger focus on how their recommendations will be audited and brought into practice and, finally d) the issue of severity is generally neglected in existing guidelines, hence there is a need for future guidelines to take this dimension into consideration and align with the forthcoming ICD-11. Collaboration between researchers working across Europe is needed to speed up this important development work and, in this respect, the ESSPD provides a helpful platform for dialogue and development on this topic and will support initiatives directly aimed at strengthening future collaboration on PD guidelines across Europe.
